# Likelihood of Dupuytren Contracture Recurrence After Limited Fasciectomy, Needle Aponeurotomy or Collagenase *Clostridium histolyticum*: Systematic Review of Prospective Data With 2- to 7-Year Follow-up

**DOI:** 10.1016/j.jhsg.2026.100979

**Published:** 2026-03-05

**Authors:** Michael Nocek, Dorothy Wakefield, Mark A. Vitale

**Affiliations:** ∗ University of Colorado School of Medicine, Anschutz Medical Campus, Aurora, CO; † ONS Foundation for Clinical Research and Education, 6 Greenwich Office Park, 40 Valley Drive, Greenwich, CT; ‡ Department of Orthopaedic Surgery, Greenwich Hospital, Yale–New Haven Health, 5 Perryridge Road, Greenwich, CT

**Keywords:** Collagenase *Clostridium histolyticum*, Dupuytren contracture, Fasciectomy, Needle aponeurotomy, Recurrence

## Abstract

**Purpose:**

Compare contracture recurrence after limited fasciectomy (LF), collagenase *Clostridium histolyticum* (CCH), and needle aponeurotomy (NA) for Dupuytren contracture (DC) using prospectively collected studies with ≥2-year follow-up and assess recurrence by joint (metacarpophalangeal [MCP] vs proximal interphalangeal [PIP]).

**Methods:**

Systematic review restricted to prospective cohort series or randomized controlled trials treating DC with LF, CCH, and/or NA. Eligibility required ≥2-year, in-person clinical follow-up and explicit definition of contracture recurrence. Exclusions included non-English publications, telephone/questionnaire-only follow-up, no recurrence data at follow-up, and sample size <15 patients. The primary end point was recurrence by treatment. Secondary analyses evaluated recurrence by joint treated and reintervention and complications when reported.

**Results:**

Ten studies met criteria, encompassing 1,411 patients and 1,698 treated joints with a mean follow-up of 3.8 years. Across studies, LF demonstrated a lower risk of recurrence than both CCH and NA, and CCH demonstrated a lower risk of recurrence than NA. Recurrence was less likely in the treatment of MCP versus PIP contractures across all treatments. For studies that reported subsequent procedures for recurrence, there was a lower likelihood of reinterventions after LF than after CCH or NA. Commonly reported complications included digital nerve injury, tendon injury, skin tears, swelling, pain, and bleeding.

**Conclusions:**

In prospectively collected cohorts with a mean 3.8 years of follow-up, recurrence after DC treatment was common. LF demonstrated the lowest recurrence (16.5%), CCH had an intermediate recurrence (32.5%), and NA showed the highest recurrence (46.4%). PIP joint contractures were more likely to recur than MCP joint contractures, regardless of treatment.

**Clinical relevance:**

LF offers greater durability and a lower likelihood of subsequent procedures than CCH or NA in the treatment of DC at intermediate term follow-up; however, the clinician must also weigh the length of recovery and potential complications between these three treatment options. Patients with PIP contractures should be counseled about a higher recurrence risk and potential need for closer follow-up.

Dupuytren contracture (DC), also known as Dupuytren disease, is a chronic fibroproliferative disorder in which pathologic nodules and cords develop on the palmar fascia of the hand with variable progression to digital flexion contractures. The prevalence of DC varies across populations, seems to be more common in individuals of northern European descent, and has been estimated to occur in approximately 2.3% of the general population.^1^ When digital contractures develop, they most commonly involve the metacarpophalangeal (MCP) and proximal interphalangeal (PIP) joints of the ring and little fingers.^2^ While nodules or mild contractures may have minimal effect on hand function, more severe contractures can impair activities of daily living and warrant treatment.^3^, ^4^, ^5^

Established treatments with demonstrated efficacy include fasciectomy, injection of collagenase *Clostridium histolyticum* (CCH), and needle aponeurotomy (NA). Fasciectomy surgically removes diseased fascia, with numerous variations including limited fasciectomy (LF), radical fasciectomy, and dermofasciectomy.^6^, ^7^, ^8^ CCH injection, Food and Drug Administration (FDA) approved in 2010,^9^ is a nonsurgical alternative that uses two enzymes that preferentially cleave type I and III collagen in Dupuytren cords, followed ≥24 hours later by manipulation into extension to rupture the enzymatically weakened cord.^10^, ^11^, ^12^ NA is a percutaneous fasciotomy technique using a hypodermic needle to puncture and partially section the cords under local analgesia, followed by immediate manipulation to rupture the mechanically weakened cords.^13^^,^^14^ Although all three methods are effective, none guarantees a permanent “cure”, and recontracture commonly occurs over time.^15^

It is challenging to counsel patients on the relative durability of these three procedures, as existing published studies are largely retrospective with small sample sizes and lower data quality. Authors have attempted to conduct systematic reviews of existing data to overcome issues related to small sample sizes; however, previous systematic reviews have had notable methodological limitations. For instance, studies included in prior systematic reviews have largely reported outcomes through 2–3-year follow-up,^16^^,^^17^ which may undercapture longer-term recurrence beyond 3 years. Many prior reviews have also included retrospective series subject to measurement and selection biases, further skewing recurrence data. Older systematic reviews did not include CCH as a treatment, as it has only been FDA approved for use since 2010.^9^ CCH was introduced in the European Union in 2011, but was revoked by the manufacturer in 2019,^18^ further limiting available data on CCH. Furthermore, existing reviews have included inconsistent or no clear definition of recurrence, further clouding results; authors have bemoaned the need for a consistent definition of DC recurrence,^17^^,^^19^, ^20^, ^21^, ^22^ and this lack of a clear definition may also in part explain why reported contracture recurrence rates vary so widely within the same treatment. One study showed that applying different definitions to a single data set changed the reported recurrence rates from 2% to 86%.^21^

We hypothesized that a rigorous systematic review of available publications limited to high-quality, prospectively collected data with a minimum 2-year follow-up would indicate that treatment with LF has the lowest likelihood of recurrence, followed by CCH and NA, and treatment of PIP joint contractures has a higher likelihood of recurrence versus MCP contractures across all treatments.

## Materials and Methods

### Study identification

This systematic review adhered to the Preferred Reporting Items in Systematic Review and Meta-Analysis (PRISMA) guidelines.^23^ A comprehensive literature search was conducted to identify studies reporting long-term outcomes of treatment for DC. The search included four databases: PubMed, Cochrane Library, Web of Science, and Google Scholar, covering articles published through December 2024. Search terms included combinations of keywords and MeSH terms: ‘Dupuytren∗’ (where ∗ denotes truncation to capture term variants), ‘Dupuytren’s contracture’, ‘Dupuytren’s disease’, ‘palmar fibromatosis’, ‘fasciectomy’, ‘fasciotomy’, ‘dermofasciectomy’, ‘aponeurotomy’, ‘aponeurectomy’, ‘needle aponeurotomy’, ‘percutaneous needle fasciotomy’, ‘percutaneous needle aponeurotomy’, ‘percutaneous fasciotomy’, ‘percutaneous aponeurotomy’, ‘collagenase’, ‘collagenase *Clostridium histolyticum*’, ‘xiaflex’, and ‘xiapex’. Reference lists of eligible articles were also reviewed to identify additional relevant studies.

### Study inclusion

**Figure 1:**
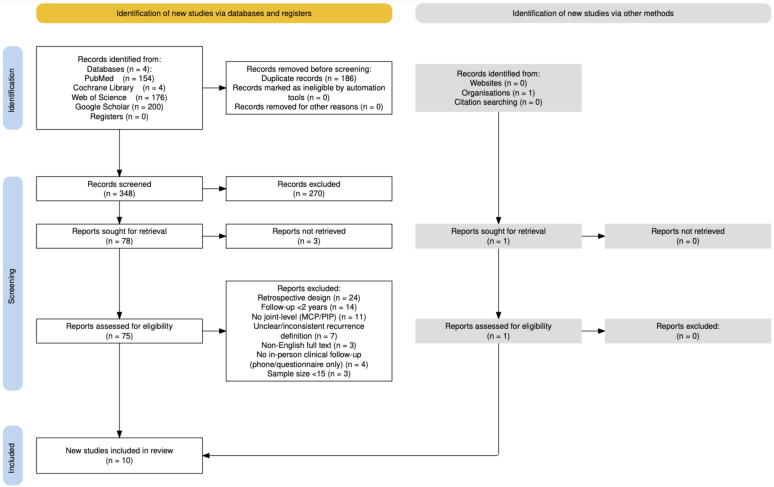
PRISMA flow diagram of study selection: diagram shows the identification, screening, and inclusion of studies, resulting in 10 eligible prospective studies. MCP, metacarpophalangeal; PIP, proximal interphalangeal.

Studies were eligible for inclusion if they met the following criteria: (1) prospective case series or randomized controlled trial (RCT) in human subjects, (2) treatment of DC with LF, NA, and/or CCH injection, (3) minimum of 2-year clinical follow-up, (4) use of a clearly defined, consistent threshold for contracture recurrence, (5) available data on recurrence outcomes at final follow-up stratified by joint (MCP or PIP), and (6) a sample size of at least 15 patients. A minimum of 2-year follow-up was chosen as contracture recurrence is relatively rare within the first year of treatment.^20^ For any studies meeting the above criteria that did report on recurrence but not by joint, authors were contacted to determine if they had joint-level recurrence data that could be further analyzed; such studies were included if the data were available. Articles not published in English, lacking recurrence definitions, without in-person clinical follow-up (eg, telephone or questionnaire follow-up only), or without reported contracture recurrence data at final follow-up were excluded. The selection process is summarized in the PRISMA flow diagram (Fig. 1).

### Data extraction

Two independent reviewers screened all titles and abstracts for relevance. Full-text review was conducted for studies meeting initial screening criteria. Discrepancies were resolved through discussion or consultation with a third reviewer. Study characteristic data were extracted from each study (Table 1). Patient demographic and treatment variables were collected, with attention to treatment modality and involvement of the MCP and/or PIP joints (Tables 2 and 3). Other less common types of digital contractures, including distal interphalangeal (DIP) joint contractures, webspace contractures, and abduction/adduction contractures, in addition to secondary deformities, including Boutonnière deformities, were not specifically analyzed. Data were also extracted and stratified by treatment modality: LF, CCH injection, and NA. The primary outcome was contracture recurrence. Secondary outcomes, including reintervention and complication rates, were extracted when available.

**Table 1:** Summary of Study Design and Key Characteristics of Included Studies

First Author (Year)	Study Design	*N* (Patients)	Intervention(s)	Mean Follow-Up (Years)	Definition of Contracture	Definition of Recurrence	Recurrence Stratified by Joint
van Rijssen (2012)^30^	RCT	93	NA, LF	5.0	≥20° deficit at MCP or PIP	≥20° at same joint	Yes
Peimer (2015)^31^	Prospective cohort	623	CCH	5.0	≥20° deficit at MCP or PIP	≥20° at previously treated joint	Yes
Skov (2017)^29^	RCT	43	CCH, NA	2.0	≥20° deficit at PIP	≥20° at same joint	Yes
van Beeck (2017)^33^	Prospective cohort	68	CCH	2.0	≥20° deficit at MCP or PIP	≥20° at same joint	Yes
Selles (2018)^27^	RCT	52	NA, LF	5.0	≥30° deficit at PIP and/or ≥20° at MCP	≥20° at same joint	Yes
Simón-Pérez (2018)^32^	Prospective cohort	71	CCH	2.3	≥20° deficit at MCP or PIP	≥20° at same joint	Yes
Abe (2020)^25^	Prospective cohort	70	CCH, NA	3.0	≥30° deficit at MCP or PIP	≥20° at same joint	Yes
De Vitis (2020)^26^	Prospective cohort	39	CCH	7.0	≥20° deficit at MCP, any deficit at PIP	≥20° at same joint	Yes
Jørgensen (2023)^28^	RCT	68	CCH, NA	3.0	≥30° deficit at MCP	≥30° at same joint	Yes
Räisänen (2024)^34^	RCT	302	NA, CCH, LF	2.0	≥20° deficit at MCP or PIP in index through small fingers	≥20° at same joint	Yes

**Table 2:** Patient Demographics

Patient Demographics	Value∗
Total no. of patients (all studies)	1,411
No. of men (%)	1,202 (85.2%)
Average age (years)	66

∗ Values are derived based on the number of patients who completed the primary end point of at least 2 years of follow-up.

**Table 3:** Total Number of Joints Treated by Procedure and Joint Type

	MCP	PIP	Total
# Joints treated by NA	235	132	367
# Joints treated by CCH	747	309	1,056
# Joints treated by LF	181	94	275
# Joints treated (all procedures)	1,163	535	1,698

When data were incomplete or ambiguous, efforts were made to contact corresponding authors for clarification. Disagreements in extracted data were resolved by consensus. Extracted data were then compiled into summary tables for descriptive and statistical analysis.

### Risk of bias

**Figure 2:**
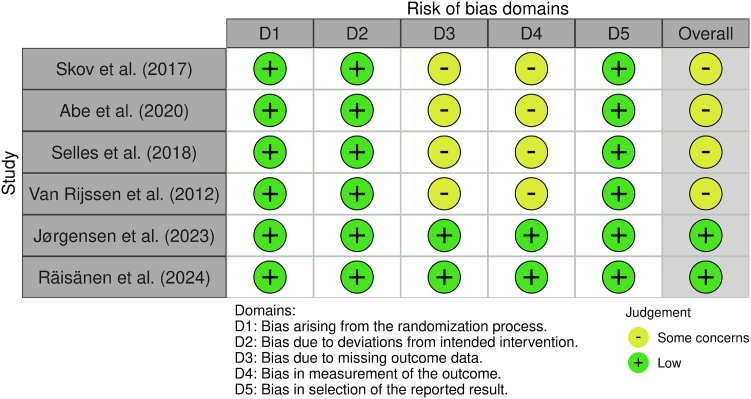
RoB 2 assessment of randomized controlled trials by bias domain (D1–D5) and overall risk. Green circles indicate low risk of bias, and yellow circles indicate some concerns.

**Figure 3:**
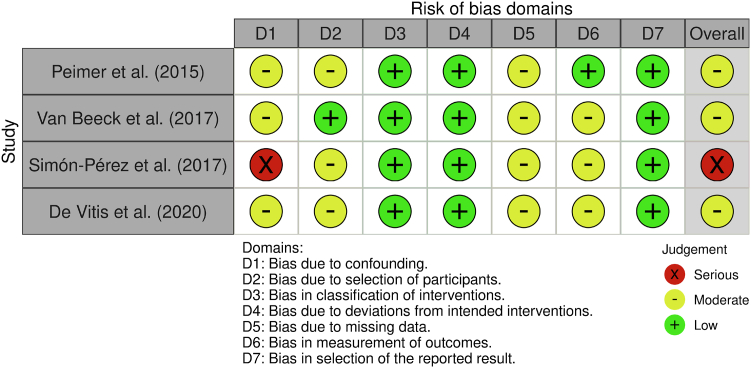
ROBINS-I assessment of nonrandomized prospective cohort studies by bias domain (D1–D7) and overall risk. Green circles indicate low risk of bias, yellow circles moderate risk, and red circles serious risk.

Two reviewers independently assessed the methodological quality of each study. RCTs were appraised with version two of the Cochrane risk-of-bias tool for randomized trials (RoB 2.0), covering five domains: randomization, deviations from intended interventions, missing data, outcome measurement, and selective reporting. Nonrandomized prospective studies were evaluated using the Risk of Bias in Non-Randomized Studies of Interventions tool (ROBINS-I), which assesses bias due to confounding, participant selection, intervention classification, deviations, missing data, outcome measurement, and reporting. Each domain was rated as low risk, some concerns, or high risk, following Cochrane Handbook guidelines. Discrepancies were resolved by consensus with a senior author. Assessments are summarized with *robvis* plots (Figs. 2 and 3).^24^

## Statistics

### Statistical analysis

The primary outcome was contracture recurrence, which was further analyzed based on treatment type and the affected joint(s) (MCP and/or PIP joint) rather than as a function of digit treated. If a patient had a contracture in a joint that was treated using any modality, that joint was included in the study. Joints that were not treated were excluded from analysis. If both the MCP and PIP joints in the same digit were affected and both were treated, then both joints were included in the study as separate data points. Recurrence was also defined on a per-joint basis. If a patient had a recurrence in only one joint, then only that specific joint was defined as having recurrence. Secondary outcomes included reintervention rate and treatment-related complications. Recurrence, joint treated, and reintervention were treated as binary outcomes for statistical analysis. Pairwise meta-analyses were conducted to compare outcomes between-treatment groups using the SAS BGLIMM procedure with a logit link function. An alpha level of 0.05 was used to determine statistical significance.

## Results

### Descriptive data

Ten prospective studies met criteria, comprising 1,411 adult patients and 1,698 joints (Table 1).^25^, ^26^, ^27^, ^28^, ^29^, ^30^, ^31^, ^32^, ^33^, ^34^ None of the 1,698 joints included had prior treatment of their contracture. LF was the only method of fasciectomy reported. One study reporting on NA added simultaneous lipofilling.^27^ No studies reported concomitant corticosteroid injection at the time of intervention. For one study in which joint-level recurrence data were unavailable in the publication, authors were contacted and provided data on recurrence as a function of joint for inclusion.^34^

All RCTs demonstrated low risk in randomization and intervention fidelity (D1–D2) but showed some concerns regarding missing data and outcome measurement (D3–D4). As a result, overall risk was rated as “some concerns” for four of six RCTs. Two RCTs achieved a “low risk” rating (Fig. 2).^28^^,^^34^ Among nonrandomized studies, moderate risk was common, particularly in confounding, selection, missing data, and outcome measurement (D1, D2, D5, D6). One study was judged to have serious overall risk due to confounding bias (Fig. 3).^32^

Demographic variables were variably reported (Table 2). Most patients underwent CCH (62.2%), followed by NA (21.6%), and LF (16.2%, Table 3). Patient/joint, follow-up time, and recurrence information were stratified by treatment (Table 4). Overall weighted mean follow-up was 3.8 years (range 2–7 years). ANOVA analysis revealed that weighted mean follow-up time (by number of patients) did not differ between groups (CCH = 4.2 years, NA = 3.4 years, and LF = 3.2 years). Joints treated with LF had the lowest recurrence rate (16.5%), followed by CCH (32.5%) and NA (46.4%).

**Table 4:** Number of Patients/Joint, Follow-Up time, and Recurrence as a Function of Treatment Type

Treatment	No. of Studies	No. of Patients, *n* (%)	No. of Joints, *n* (%)	Follow-Up Years, Median (Min, Max)	Recurrence Rate (Min, Max)
NA	6 (Study: A, B, C, D, E, and J)	267 (18.9)	367 (21.6)	3 (2, 5)	46.4 (18, 79)
CCH	8 (Study: B, C, D, F, G, H, I, and J)	985 (69.8)	1,056 (62.2)	3 (2,7)	32.4 (5, 92)
LF	3 (Study: A, E, and J)	159 (11.3)	275 (16.2)	5 (5, 5)	16.5 (5, 39)
Total	9 (Study: A–I)	1,411	1,698	4 (2, 7)	

The likelihood of recurrence varied among treatment groups (Table 5). Mixed model analyses showed that joints treated with CCH had a significantly lower likelihood of recurrence compared to NA (OR = 0.64, CI 0.42, 0.98) but had a higher likelihood of recurrence compared to joints treated with LF (OR = 2.7, CI 1.20, 5.99). Joints treated with NA also had a significantly higher likelihood of recurrence compared to LF (OR = 4.55, CI 2.65, 7.81). Across all three treatments, MCP contractures were two-thirds less likely to recur than PIP contractures (OR = 0.34, CI: 0.27, 0.44).

**Table 5:** Likelihood of Contracture Recurrence as a Function of Treatment and Joint Treated

Comparison	Odds Ratio	Confidence Interval
CCH vs NA	0.64	0.42–0.98
CCH vs LF	2.71	1.20–5.99
NA vs LF	4.55	2.64–7.81
MCP vs PIP	0.34	0.27–0.44

Seven studies reported on reinterventions. For LF, 5 of 13 (38.5%) recurrences had a reintervention (2 studies); for NA, 13 of 42 (31%) recurrences had a reintervention (2 studies); for CCH, 146 of 367 (39.8%) recurrences had a reintervention (5 studies). Joints treated with LF had the lowest reintervention rate (1.8%), followed by NA (3.5%) and CCH (13.8%, Table 6). Mixed model analyses showed that joints treated with CCH were nine times more likely to have reintervention than LF (OR 9.15, CI 2.07, 44.88), and joints treated with NA were more than seven times more likely to have reintervention than LF (OR = 7.68, CI 1.6, 41.00, Table 7). The likelihood of reintervention was not significantly different in joints treated with CCH versus NA.

**Table 6:** Reintervention Rates and Number of Joints Requiring Reintervention by Procedure

Procedure	Total Joints Treated	No. of Joints Requiring Reintervention (%)
NA	367	13 (3.5%)
CCH	1056	146 (13.8%)
LF	275	5 (1.8%)

**Table 7:** Likelihood of Reintervention as a Function of Treatment

Comparison	OR	CI
CCH vs NA	1.43	0.65–3.08
CCH vs LF	9.15	2.07–44.88
NA vs LF	7.68	1.60–41.00

The most common complications were digital nerve injury, tendon injury, skin tear, swelling, pain, and bleeding. Data on complications are summarized (Table 8). Statistical comparisons of complications across treatments and joints were not performed as the data on complications were of high heterogeneity in reporting across studies, precluding meaningful comparisons.

**Table 8:** The Ten Most Reported Complications Across All Studies

Complication	No. of Studies Reporting
Digital nerve injury	6
Skin tear	4
Pain and swelling	4
Bleeding	4
Flexor tendon injury	3
Allergic reaction	2
Infection/wound healing	2
Complex regional pain syndrome	2
Hyperesthesia	1
Stiffness	1
No complications reported	2

Because complications were inconsistently reported and often not stratified by treatment modality, this table reflects the number of studies reporting each complication rather than incidence or modality-specific frequency.

## Discussion

DC is often described as a condition without a “cure”, as contracture recurrence remains common after treatments. The challenge of recurrence has stimulated investigation of alternative therapies to minimize disease progression and/or reduce the likelihood of recurrence.^35^^,^^36^ The decision on treatment modality is often made without a clear understanding of what recurrence risk is for a given treatment. Attempts have been made to systematically review the likelihood of recurrence after treatments, but four systematic reviews conducted before 2019 had methodological flaws of retrospective data, short follow-up, small sample sizes, and no objective definition of recurrence, as well as limited inclusion of CCH.^17^^,^^20^^,^^37^^,^^38^

More recent (2023–2025) systematic reviews have attempted to address these flaws, but all had notable limitations. A 2023 meta-analysis of RCTs with ≥3-year follow-up (*n* = 347; CCH vs NA) found no differences in recurrence or final flexion contracture, but LF was not evaluated.^39^ A subsequent 2024 systematic review (11 studies; *n* = 1,051) reported higher recurrence rate with CCH (25.8%) versus LF (9.3%) and more severe complications with LF (7.3%) versus CCH (0.3%), but included short follow-up (≥3 months) and nonstandard recurrence definitions (two studies used patient perception of need for reintervention).^40^ A 2025 systematic review of RCTs (11 trials; *n* = 969) comparing CCH, NA, and LF found no difference in recurrence between CCH and NA, but higher recurrence in CCH versus LF. Interpretation was limited by short-term follow-up (minimum 12 months) and heterogeneous recurrence definitions (eg, counting reintervention as recurrence).^41^ Another 2025 meta-analysis (15 studies) used time-to-event methods and reported the highest recurrence after CCH (41%), followed by NA (33%) and LF (10%) at 3-year median follow-up. Time-to-recurrence did not differ between CCH and NA, whereas younger age and female sex predicted earlier recurrence. Recurrence analyses, however, included only 245 patients (CCH 66, LF 51, NA 128), and 9 of 15 studies were retrospective, limiting power and internal validity.^42^ The most recent 2025 meta-analysis of 17 RCTs (*n* = 1,010) showed LF had the lowest long-term recurrence compared to NA and CCH but had more severe complications; this study was limited by shorter follow-up (max = 3 years) and no explicit recurrence definition.^16^

The present review addressed these limitations by enforcing a strict recurrence definition, including only prospective studies and requiring ≥2-year follow-up. Among 1,411 patients (1,698 joints), recurrence risk ranked LF < CCH < NA: CCH had approximately one-third lower odds of recurrence than NA; CCH had approximately threefold higher odds of recurrence than LF; NA had roughly fivefold higher odds of recurrence than LF. A mechanistic rationale for this observation is that LF removes the largest burden of diseased tissue, CCH enzymatically degrades cords, removing some burden of disease, and NA divides tissue without removal. Prior reviews consistently found higher recurrence after CCH or NA than LF; to our knowledge, this is the first demonstration of higher recurrence after NA versus CCH.

Furthermore, the current review revealed that, regardless of treatment, the risk of recurrence was two-thirds less likely for MCP contractures compared to PIP contractures at a mean follow-up of 3.8 years, consistent with previous studies. For instance, one prospective cohort study found recurrence rates of 14% for MCP joints and 23% for PIP joints following CCH.^43^ Similarly, a 10-year follow-up study reported recurrence rates of 54.8% for MCP joints and 100% for PIP joints after CCH.^44^ These findings suggest that PIP joints are more susceptible to recurrence, regardless of the treatment. This may be partially explained by combined volar plate/collateral ligament contracture with extensor mechanism attenuation in chronic PIP disease. Patient characteristics may influence recurrence, including family history, alcohol use, age at onset, body mass index, and sex.^45^ Our analysis did not adjust for these variables, and some included nonrandomized studies may reflect confounding by indication.

A secondary end point of this study was the likelihood of reintervention after initial treatment. Seven of the 10 included studies reported on reinterventions. Patients treated with CCH were nine times more likely to have reintervention than those treated with LF, and patients treated with NA were over seven times more likely to have reintervention than those treated with LF. Several factors may influence the likelihood of reintervention independently of contracture recurrence, including perceptions that surgery offers more permanent results and the desire to avoid pain, recovery time, or inconvenience associated with specific interventions. Moreover, the potential for severe surgical complications may contribute to a diminished willingness to undergo repeat surgery. Interestingly, although CCH had a lower recurrence risk than NA, it did have a higher reintervention risk for reasons that are unclear. One potential explanation is that not all reinterventions were necessarily for recurrence. For instance, a joint treated with CCH that resulted in tendon rupture may have been captured as a reintervention; in this case, the reintervention would have been to treat a complication rather than a recurrence.

The most common complications identified in this review were digital nerve injury, tendon injury, skin tears, swelling, pain, and bleeding. The data on complications in the present review were heterogenous and reported inconsistently, precluding meaningful statistical analysis between groups. While the incidence of severe complications was generally low with first-time surgical treatment,^25^^,^^29^^,^^30^ the incidence of severe complications has been reported to be much higher with repeat fasciectomy or dermofasciectomy.^46^ Ideally, treatments for DC should be chosen not only based on their ability to delay/prevent recurrence but also based on risk of complications and expected recovery time.

This review has limitations. Although we required a ≥2-year follow-up, several studies still had relatively short observation. Long-term surveillance is costly, and emerging biomarkers may offer shorter-horizon surrogates of disease activity before recurrent contractures are evident.^47^ The LF cohort was relatively small (275/1,698 joints), reducing power for between-treatment comparisons. We sought to apply a uniform, objective recurrence definition, but one study^28^ had a slightly different definition than the other nine, introducing heterogeneity. A consensus definition remains unsettled: one group defined recurrence as passive extension deficit >20° in ≥1 joint with a palpable cord compared with 6–12 weeks presurgery,^21^ whereas a Delphi panel endorsed >20° contracture recurrence in any treated joint at 1-year post-treatment relative to 6 weeks post-treatment.^19^

Future studies should aim to address these limitations by employing longer follow-up periods to capture long-term recurrence data, ensuring more balanced representation between-treatment groups and standardizing treatment protocols and definitions of recurrence to best understand the risks of contracture recurrence after treatments. Nevertheless, the current data may help inform clinicians and patients on the relative likelihood of recurrence after each of these three common treatments.

## Conflicts of Interest

Dr. Mark A. Vitale served on the advisory board for Endo Pharmaceuticals, Inc in 2022. The other authors have no relevant conflicts to disclose.
